# MicroRNA-302a Suppresses Tumor Cell Proliferation by Inhibiting *AKT* in Prostate Cancer

**DOI:** 10.1371/journal.pone.0124410

**Published:** 2015-04-29

**Authors:** Gui-Ming Zhang, Chun-Yang Bao, Fang-Ning Wan, Da-Long Cao, Xiao-Jian Qin, Hai-Liang Zhang, Yao Zhu, Bo Dai, Guo-Hai Shi, Ding-Wei Ye

**Affiliations:** 1 Department of Urology, Fudan University Shanghai Cancer Center, Shanghai, China; 2 Department of Oncology, Shanghai Medical College, Fudan University, Shanghai, China; 3 State Key Laboratory of Oncogenes and Related Genes, Shanghai Cancer Institute, Ren Ji Hospital, Shanghai Jiao Tong University School of Medicine, Shanghai, China; Sun Yat-sen University Medical School, CHINA

## Abstract

Micro (mi) RNAs are important regulators involved in various physical and pathological processes, including cancer. The miRNA-302 family has been documented as playing a critical role in carcinogenesis. In this study, we investigated the role of miRNA-302a in prostate cancer (PCa). MiRNA-302a expression was detected in 44 PCa tissues and 10 normal prostate tissues, and their clinicopathological significance was analyzed. Cell proliferation and cell cycle analysis were performed on PCa cells that stably expressed miRNA-302a. The target gene of miRNA-302a and the downstream pathway were further investigated. Compared with normal prostate tissues, miRNA-302a expression was downregulated in PCa tissues, and was even lower in PCa tissues with a Gleason score ≥8. Overexpression of miRNA-302a induced G1/S cell cycle arrest in PCa cells, and suppressed PCa cell proliferation both in vitro and in vivo. Furthermore, miRNA-302a inhibits *AKT* expression by directly binding to its 3΄ untranslated region, resulting in subsequent alterations of the *AKT-GSK3β-cyclin D1* and *AKT-p27^Kip1^* pathway. These results reveal miRNA-302a as a tumor suppressor in PCa, suggesting that miRNA-302a may be used as a potential target for therapeutic intervention in PCa.

## Introduction

As the most prevalent malignancy among men in developed countries [1], prostate cancer (PCa) likewise shows a steady rise in incidence in China over the past few decades. According to the Chinese Cancer Registry Annual Report (2012), PCa has become the sixth most common cancer and the ninth leading cause of cancer-related mortality in men, especially in urban areas [2]. Additionally, up to 70% of patients with PCa have metastases at the time of diagnosis, resulting in dramatically decreased long-term survival [3]. Hence, there is an imperative need to explore the mechanisms by which PCa generation and progression are initiated.

Growing evidence indicates that microRNAs (miRNAs), a type of endogenous, small noncoding RNAs, participate in diverse cellular processes. Through specifically binding and cleaving mRNAs or inhibiting their translation [4,5], miRNAs function as either oncogenes or tumor suppressors [6]. The miRNA-302 family was first identified in human embryonic stem cells (hESCs) and human embryonic carcinoma cells in 2004. Since then, various studies on the miRNA-302 family have focused on its potential role in reprograming somatic cells into induced pluripotent stem cells, as well as embryonic self-renewal [7]. Several transcription factors that are expressed early in cancer stem cell development and maintenance, such as *Oct4*, *Sox2*, and *Nanog*, were found essential for the transcriptional regulation of the miRNA-302 family [8]. Interestingly, Fareh et al. demonstrated that stable expression of miRNA-302 was able to induce loss of *Oct4*, *Sox1*, and *Nanog* [9]. The role of miRNA-302 in tumorigenesis has been debated recently, as conflicting conclusions have been drawn by different research groups. For instance, endogenous miRNA-302 was not detected in cervical cancer cells, and ectopic expression of miRNA-302 inhibited cell proliferation and tumor formation [10]. In contrast, transfection of miRNA-302b in colon cancer cells resulted in an increased ability for colony-formation, invasion, and migration in vitro [11]. However, to date, few studies have been conducted to investigate the possible role of miRNA-302 in PCa.

In the present study, we found that, compared with normal prostate tissues, PCa tissues expressed lower miRNA-302a levels, and miRNA-302a expression was inversely associated with Gleason score (GS). We also show that overexpression of miRNA-302a in PCa cells can induce cell cycle arrest and inhibit cell proliferation in vitro and tumor formation in vivo. In addition, we identified *AKT* as a target gene through which miRNA-302a exerts its inhibitory role in PCa.

## Materials and Methods

### Patient samples

PCa tissue and benign prostate tissue were obtained from the tissue bank at Fudan University Shanghai Cancer Center. Clinicopathological features of these patients were retrieved from the Department of Urology database. The study protocol was approved by the Institutional Research Review Board at Fudan University Shanghai Cancer Center and signed informed consent was obtained from all study participants.

### Cell culture

Human PCa cell lines, human embryonic kidney 293T cells (HEK293T) and normal prostate epithelial cells (RWPE-1) were purchased from the Institute of Cell Research of the Chinese Academy of Sciences (Shanghai, People’s Republic of China). LNCaP and 22Rv1, PC-3, DU145, and HEK293T cells were grown in RPMI 1640 medium, F-12K medium, MEM medium, and DMEM medium, respectively, all supplemented with 10% fetal bovine serum. RWPE-1 cells were grown in K-SFM medium supplemented with bovine pituitary extract and human recombinant epidermal growth factor. Cells were cultured at 37°C at 5% CO_2_.

### RNA, miRNA extraction, and quantitative real-time polymerase chain reaction

Total RNA was isolated from cultured cells and tumor tissues using Trizol reagent. First strand cDNA was synthesized using the RevertAid First Strand cDNA synthesis Kit (Life technology, Carlsbad, CA), which was then used for real-time polymerase chain reaction (PCR), together with forward and reverse primers and the Power SYBR Green PCR Master Mix. *β-actin* was used as an internal control for *AKT* transcript levels. The primer sequences were as follows: *AKT*-forward: GGGTTTCTCCCAGGAGGTTT, reverse: GTCCATGGTGTTCCTACCCA; β-actin-forward: ACCGAGCGCGGCTACAG, reverse: CTTAATGTCACGCACGATTTCC.

According to the manufacturer’s instructions, miRNA from tissues and cells was extracted using the mirVana miRNA isolation kit (Life technology, Carlsbad, CA), and the expression levels of miRNA-302a were detected by TaqMan miRNA assays (Life technology, Carlsbad, CA), using U6 small nuclear RNA as an internal control.

### Vector construction, lentivirus production, and cell transfection

The mature hsa-miRNA-302a sequence was synthesized and introduced into the PLKO.3G vector to produce PLKO.3G-miR-302a. An AKT restoration vector was constructed by introducing the *AKT* CDS which was amplified from PC-3 cDNA into the pCDH-CMV-MCS-EF1-copGFP vector. The luciferase-3΄ untranslated region (UTR) reporter vector was generated through constructing the *AKT* 3΄UTR, which carries a putative miRNA-302a binding site into vector MT01. All the constructed vectors were verified by sequencing.

PLKO.3G-miR-302a mixed with psPAX2 and PMD2-G was transfected into HEK293T cells using lipofectamine 2000 reagent (Invitrogen, Carlsbad, CA) according to the manufacturer’s protocol. Forty-eight hours later, lentivirus was harvested and used to infect PC-3 and DU145 cells. Next, the cells were sorted by flow cytometry (Beckman Coulter, Brea, CA) to establish stable cell lines constitutively expressing miRNA-302a (PC-3-302a and DU145-302a cells).

### Luciferase assays

Forty-eight hours after transfection, cells were lysed using 50 μL of passive lysis buffer. Next, a dual-luciferase assay was carried out as directed by the manufacturer (Promega, Madison, WI). The ratio of firefly to Renilla luciferase activity was used to express luciferase activities. All experiments were performed in triplicate.

### Protein harvest and western blot

Total proteins were harvested using the CelLytic Extraction kit (Roche, Basel, Switzerland) containing protease inhibitors and then quantified using the BCA Protein Assay Reagent kit (Thermo Fisher Scientific, Waltham, MA) according to the manufacturer's instructions. After separating proteins using sodium dodecyl sulfate polyacrylamide gel electrophoresis, protein was transferred to polyvinylidene fluoride membranes and then blocked in 5% defatted milk. Using the primary antibodies and anti-rabbit linked to horseradish peroxidase (1:5000) (Santa Cruz Biotechnology, Dallas, TX) as the second antibody, the target proteins were probed and then visualized using the ECL PlusWestern Blotting System (Thermo Fisher Scientific, Waltham, MA). *β-actin* was use as a loading control. The primary antibodies included the following: *AKT* (1:1000), phosphorylated *AKT* (*pAKT*)*(Ser473)* (1:500), *GSK3β* (1:500), *pGSK3β(Ser9)* (1:500), *cyclin D1* (1:1000), *p27* ^*Kip1*^ (1:1000), *PI3K* (1:1000), (Cell Signaling Technology, Boston, MA) and *β-actin* (1:2000) (Santa Cruz Biotechnology, Dallas, TX).

### Cell proliferation and cell cycle assays

CCK-8 and EdU assays were performed to detect cell proliferation. Briefly, CCK-8 assays were carried out as follows: cells were seeded in a 96-well plate at a concentration of 1 × 10^4^ cells/well. After adherence, the cells were cultured in fresh medium mixed with CCK-8 (10:1) (Dojindo, Shanghai, China) for 2 hours, before the absorbance was measured with a microplate reader at 450 nm. For EdU assays, cells were incubated in EdU solution (1:5000) for 2 hours, then were harvested and stained using the Cell-Light EdU Apollo 643 In vitro Flow Cytometry Kit (Ribobio, Guangzhou, China), according to the manufacturer’s instructions. The cells were then analyzed by flow cytometry.

A cell cycle assay was also performed using flow cytometry: briefly, cells were fixed with 75% cold ethanol overnight, and then washed with phosphate-buffered saline. Next, propidium iodide (50 μg/mL) containing RNase was added to the cells for DNA staining before flow cytometry analysis.

### Colony formation assay

Isolated cells were seeded in 60 mm plates at a concentration of 500 cells/well and then incubated in 5% CO_2_ at 37°C. Twenty days later, cells were stained with 0.5% crystal violet for 30 minutes. Colony numbers in each plate were counted using an inverted microscope.

### In vivo tumorigenicity

The PLKO.3G-Scr-transfected PC-3 cells (PC-3-Scr cells) and PC-3-302a cells were injected subcutaneously into either posterior flank of the same 4–6-week-old male BALB/c nude mouse, which were purchased from Shanghai SLAC Laboratory Animals Co., Ltd. (Shanghai, China). Tumor sizes were measured using calipers at least three times weekly. The mice were euthanized with CO_2_ on day 44. Tumor volume was calculated and tumor weight was measured after sacrifice. Tumors were then divided into two parts, each part fixed with 10% formalin or preserved in −80°C. The animal experiments were performed with the approval of the Animal Studies Ethics Committee of Fudan University Shanghai Cancer Center.

### Immunohistochemistry

Immunohistochemistry (IHC) staining of paraffin-embedded specimens was performed as previously described [12]. Briefly, rabbit anti-*AKT* antibody and anti-mouse/rabbit horseradish peroxidase-labeled antibody (Univ-bio, Shanghai, China) were used as the primary and the second antibody, respectively.

### Statistical analyses

The difference between continuous variables was analyzed using the Student’s *t*-test or analysis of variance. Two-sided *P*-values < 0.05 were considered statistically significant. Statistical analyses were performed using SPSS version 20.0 (IBM Corporation, NY).

## Results

### MiRNA-302a expression is suppressed in PCa tissues and is lower with higher GS

PCa tissues were acquired from a total of 44 male patients with an average age of 67 years (range, 49 to 77 years) with newly diagnosed, pathologically confirmed PCa. Among them, 32 patients had received radical prostatectomy and 12 patients had received transurethral resection of the prostate. Pathologically confirmed normal prostate tissues were acquired from 10 male patients with bladder cancer who had received radical cystectomy. The clinical and pathological features of all patients are detailed in S1 Table.

**Fig 1:**
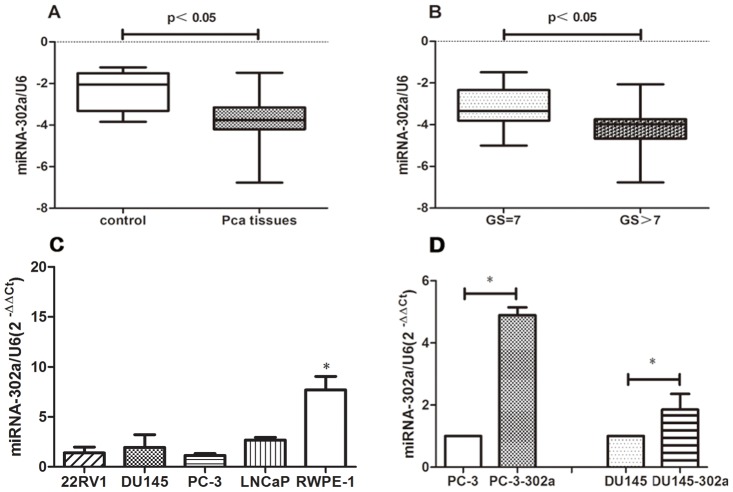
MiRNA-302a expression profiles in PCa tissue, normal prostate tissue, PCa cells and normal prostate epithelial cells. **(A)** MiRNA-302a expression was downregulated in PCa tissue compared with normal prostate tissue. **(B)** MiRNA-302a expression was lower in PCa tissues with GS > 7 compared with those with GS = 7. **(C)** Lower levels of miRNA-302a expression were detected in four human PCa cell lines compared with normal prostate epithelial cells. **(D)** High levels of miRNA-302a expression were detected in PCa cells stably expressing miRNA-302a (*P<0.05).

We detected miRNA-302a expression levels in 44 PCa tissues and 10 normal prostate tissues and found that, compared with normal prostate tissues, PCa tissues expressed lower levels of miRNA-302a (Fig 1A). Furthermore, we analyzed the relationship between miRNA-302a levels and clinicopathological features in PCa patients. There was no significant association observed between miRNA-302a levels and age, prostate-specific antigen levels, or clinical stage (S1 Table). However, comparison of miRNA-302a expression levels in different PCa tissues revealed that the expression of miRNA-302a significantly decreased when GS >7 (Fig 1B). Collectively, we postulate that downregulation of miRNA-302a expression may play an important role in PCa progression.

### Overexpression of miRNA-302a inhibits PCa cell growth in vitro and in vivo

To investigate the function of miRNA-302a in PCa, we measured the expression of miRNA-302a in four human PCa cell lines (LNCaP, 22Rv1, PC-3, and DU145) and normal prostate epithelial cells (RWPE-1) by quantitative real-time PCR. As shown in Fig 1C, there was lower expression of miRNA-302a in all four cell lines compared with RWPE-1 cells. Because we speculated that overexpression of miRNA-302a may inhibit PCa cell growth, we stably overexpressed miRNA-302a in PC-3 and DU145 cells, which was confirmed by quantitative reverse-transcriptase (qRT)-PCR (Fig 1D).

**Fig 2:**
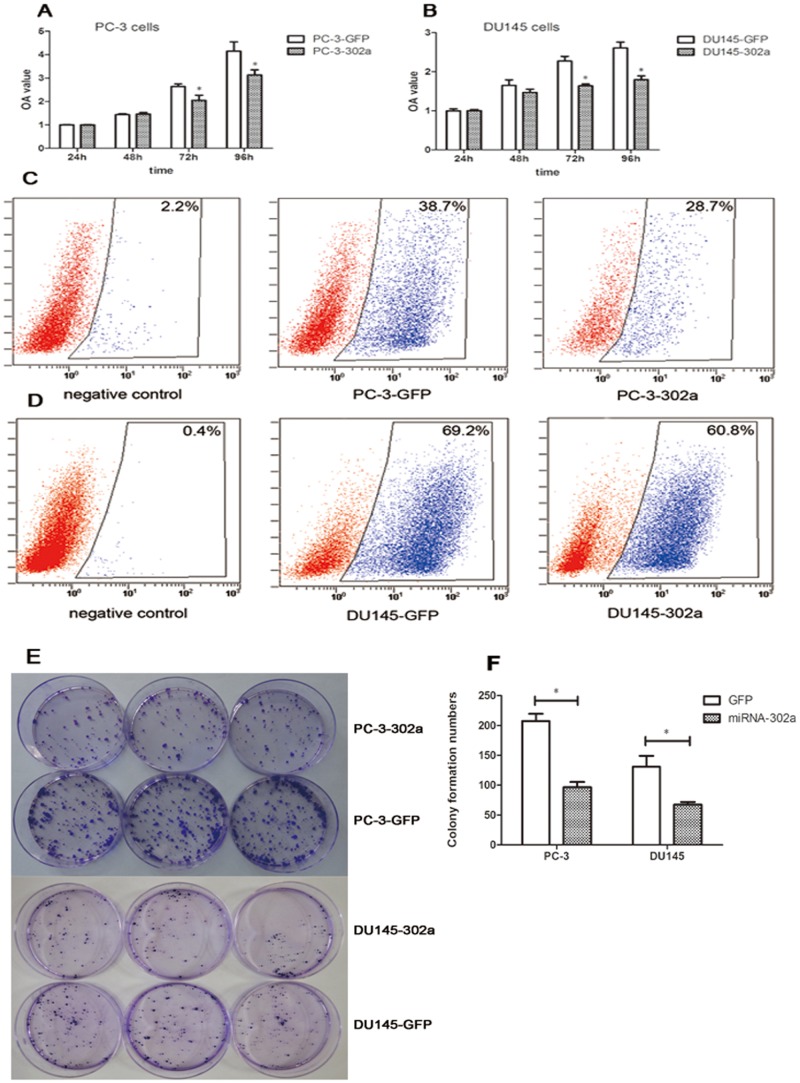
Overexpression of miRNA-302a significantly inhibits cell proliferation in PCa cells in vitro. A CCK-8 assay was performed to measure proliferation in **(A)** PC-3 and **(B)** DU145 cells. Data represent the mean ± standard deviation of the optical density (OD) value detected at 450 nm from three independent experiments. Cell proliferation was detected in **(C)** PC-3 and **(D)** DU145 cells using EdU assay analyzed by flow cytometry. **(E, F)** Colony formation assays indicated fewer colonies in miRNA-302a overexpressing PCa cells. (*P<0.05).

The CCK-8, EdU, and colony forming assays were carried out to examine whether miRNA-302a overexpression affected PCa cell proliferation in vitro. As shown in Fig 2, there was a significantly lower (*P* < 0.05) growth rate in PC-3-302a and DU145-302a cells compared with the controls. Flow cytometric analyses indicated that the percentages of EdU-positive cells in both PC-3-302a and DU145-302a cells were lower than in the controls. In addition, compared with the controls, both PC-3-302a and DU145-302a cells developed fewer colonies on the 20th and 15th days, respectively. Therefore, in vitro experiments demonstrate that miRNA-302a exerted a suppressive role in PCa cell proliferation.

**Fig 3:**
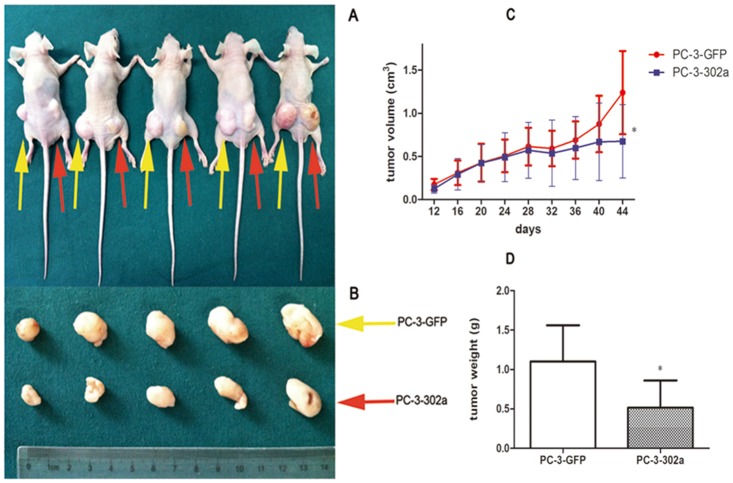
Overexpression of miRNA-302a significantly inhibits cell proliferation in PCa cells in vivo. PC-3-GFP cells and PC-3-302a cells were injected into the left and right posterior flank of BALB/c nude mice, respectively **(A, B)**. The tumor volume **(C)** and mass **(D)** in the PC-3-302a group were notably lower than in the PC-3-GFP group. (* *P*<0.05).

To further validate our observations in vivo, PC-3-Scr cells and PC-3-302a cells were injected into the left and right posterior flank of five nude mice, respectively. Tumor volumes were measured using calipers at different time points after inoculation, and tumor weights were measured after sacrifice. Both volume and mass were notably lower in PC-3-302a tumors than in PC-3-Scr tumors (*P* < 0.05; Fig 3). Taken together, obvious cell proliferation inhibition was observed after overexpression of miRNA-302a in PCa cells.

### Overexpression of miRNA-302a induces cell cycle arrest in PCa cells

**Fig 4:**
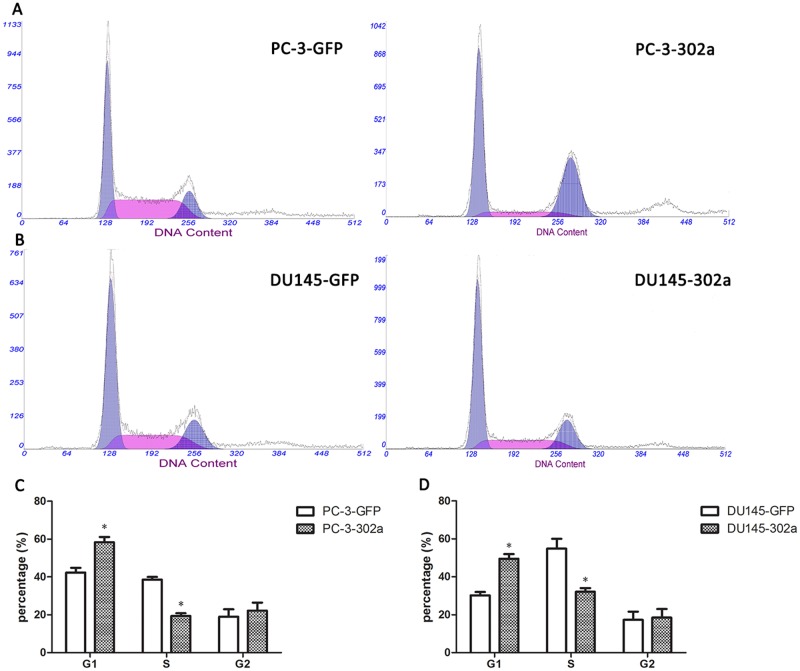
Overexpression of miRNA-302a induces G1/S phase cell cycle arrest in PCa cells. The proportion of cells in G1-phase increased significantly in PC-3-302a cells **(A, C)** and DU145-302a cells **(B, D)** compared with controls, while the proportion of cells in the S phase were notably less than the controls. (* P<0.05).

Now that growth inhibition was observed in PCa cells, we performed cell cycle analysis to investigate whether overexpression of miRNA-302a resulted in cell cycle alterations. As shown in Fig 4, the proportion of cells in G1-phase increased remarkably in PC-3-302a cells, while the proportion of cells in the S phase were notably less than in the control. Analogous results were observed in DU145-302a cells, suggesting that miRNA-302a effectively induce G1/S cell cycle arrest in PCa cells.

### MiRNA-302a suppresses *AKT* expression by directly targeting its 3΄UTR

**Fig 5:**
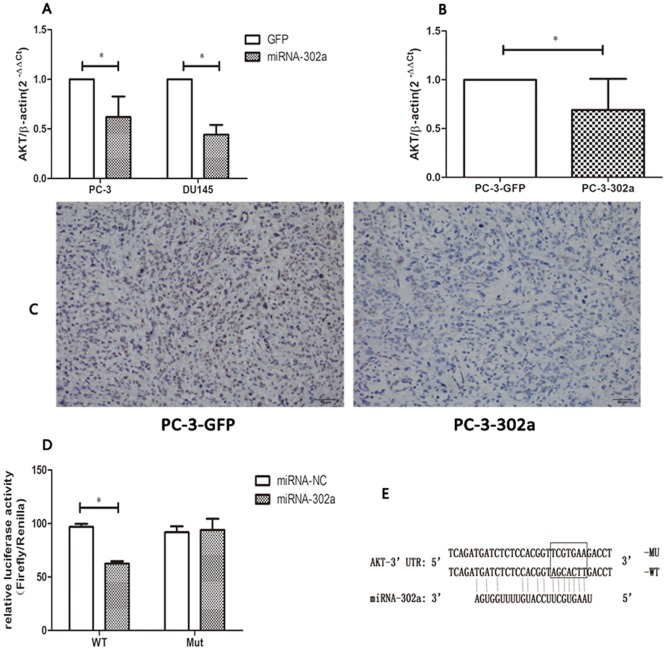
MiRNA-302a suppresses *AKT* expression by directly targeting its 3΄ untranslated region. *AKT* mRNA expression was remarkably suppressed in **(A)** PC-3-302a and DU145-302a cells, and **(B)** PC-3-302a tumors (five independent tumors were detected). **(C)** Immunohistochemistry staining indicated lower expression of *AKT* in PC-3-302a tumors. **(D)** Relative luciferase activity was notably suppressed in wild-type *AKT*-3΄ untranslated region (UTR) transfected cells. **(E)** Schematic representation of the luciferase reporter, which carried the wild-type or mutant *AKT*-3’ UTR. (* P<0.05).

**Fig 6:**
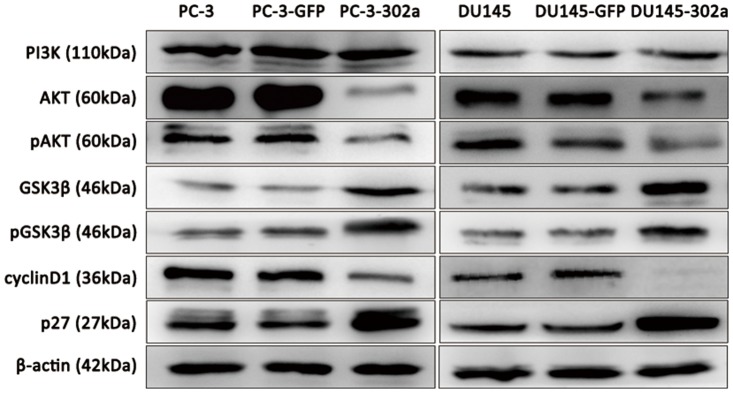
Overexpression of miRNA-302a in PCa cells triggers alterations in the *AKT-GSK3β-cyclin D1* and *AKT-p27* ^*Kip1*^signaling pathways. Western blot analyses showed downregulated AKT, phosphorylated AKT (pAKT) and cyclin D1 levels, and upregulated GSK3β,pGSK3β and p27^Kip1^ levels in miRNA-302a overexpressing PCa cells. PI3K levels were not affected.

To detect the molecular mechanisms by which miRNA-302a exerts its posttranscriptional regulatory functions, we used bioinformatics algorithms (http://www.targetscan.org) to seek possible target genes, and found that the 3΄UTR of *AKT* mRNA harbors a conserved binding site for miRNA-302a. Next, we examined the expression of *AKT* at the mRNA and protein level in PC-3-302a and DU145-302a cells and controls. As shown in Figs 5A and 6, compared with controls, *AKT* expressions decreased significantly in PC-3-302a and DU145-302a cells, at both the mRNA and protein level. Furthermore, *AKT* expression in PC-3-302a tumors was significantly lower than in PC-3-Scr tumors, as determined by real-time PCR and IHC staining (Fig 5B and 5C). These results suggest that *AKT* expression is downregulated by miRNA-302a in PCa.

To test whether miRNA-302a regulates *AKT* by directly binding to its 3΄UTR, a luciferase reporter vector containing the human *AKT* 3΄UTR that carried the wild-type miRNA-302a binding sequence was subcloned. A luciferase vector carrying the mutant 7-bp region complementary to the 5΄ seed region of miRNA-302a was generated as the control reporter (Fig 5E). Compared with the control, the relative luciferase activity was suppressed by 36% (*P* < 0.05) in wild-type *AKT* 3΄UTR transfected cells (Fig 5D). Therefore, miRNA-302a inhibits PCa cells via directly targeting *AKT*.

### MiRNA-302a induced cell cycle alterations in PCa cells by inhibiting the *AKT-GSK3β-cyclin D1* and *AKT-p27* ^*Kip1*^pathways

**Fig 7:**
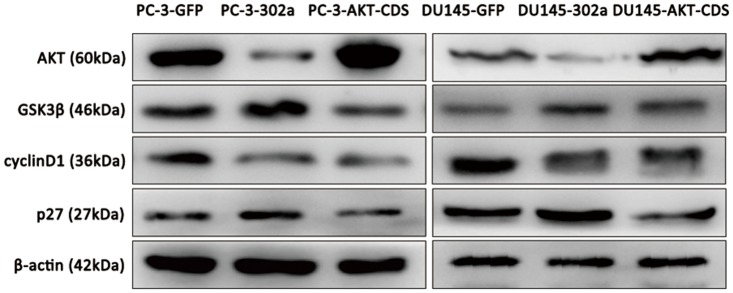
Alterations of AKT-GSK3β-cyclin D1 and *AKT-p27* ^*Kip1*^ signaling pathways after *AKT* restoration in miRNA-302a overexpressed PCa cells. Western blot analyses showed rescued expression of AKT, and inhibited expression of GSK3β and p27^Kip1^ levels by AKT restoration. However, cyclin D1 levels were not rescued.

To further evaluate the effect of miRNA-302a on the *AKT* signaling pathway, we detected the expression of upstream (*PI3K*) and downstream (*GSK3β*, *cyclin D1* and *p27* ^*Kip1*^) *AKT* effectors by western blot. In addition, levels of phosphorylated AKT (pAKT) and pGSK3β were also examined. Compared with the corresponding control cells, both GSK3β and pGSK3*β* levels, as well as p27^Kip1^ levels, were notably elevated, while pAKT and cyclin D1 were notably reduced in miRNA-302a transfected PC-3 and DU145 cells. However, expression of *PI3K* was not influenced by miRNA-302a transfection (Fig 6). To further confirm these findings, we restored *AKT* expression by transient transfection of an *AKT* expression vector carrying *AKT* CDS into PC-3-302a and DU145-302a cells (named PC-3-AKT-CDS and DU145-AKT-CDS respectively). As indicated in Fig 7, after restoration of AKT expression, both GSK3β and p27^Kip1^ levels were reduced, while cyclin D1 expression was not rescued in both PC-3-AKT-CDS and DU145-AKT-CDS cells. Given the critical roles of the *AKT-GSK3β-cyclin D1 and AKT-p27* ^*Kip1*^ signaling pathways in cell cycle transition, our results suggest that miRNA-302a might induce G1/S cell cycle arrest in PCa cells by simultaneously inhibiting the *AKT-GSK3β-cyclin D1* and the *AKT-p27* ^*Kip1*^ pathway, thereby suppressing PCa cell proliferation.

## Discussion

In this study, we observed that miRNA-302a was downregulated in PCa tissues. Further analyses revealed lower expression in PCa tissues with GS ≥8 than those with GS <8. Overexpression of miRNA-302a induced G1/S arrest in PCa cells, and remarkably suppressed PCa cell proliferation in vitro and in vivo. Moreover, *AKT* was revealed as a direct and functional target gene of miRNA-302a.

Over the past decade, most research involving miRNA-302 has focused on its role in hESCs, which are characterized by self-renewal and pluripotency. Studies analyzing miRNA-302 function in carcinogenesis are limited and inconsistent. Lin et al. found that miRNA-302 could inhibit tumorigenicity and induce apoptosis in human breast cancer MCF7 cells, embryonal teratocarcinoma Tera-2 cells, and hepatocellular carcinoma HepG2 cells [13]. Similarly, cell proliferation was suppressed in cervical cancer Hela and SiHa cells, as well as hepatocellular carcinoma SMMC-7721 cells, via cell cycle regulation [10,14]. However, Zhu et al. observed an inverse phenomenon in colon cancer cells: overexpression of miRNA-302b led to enhanced colony-forming ability in vitro [11]. The augmented colony formation ability was likewise validated in cancer stem cells from head and neck squamous cell carcinoma [15]. For the first time, we revealed suppressed miRNA-302a expression in PCa tissues compared with normal prostate tissues, and lower expression in PCa tissues with higher GS. Hence, we speculate that miRNA-302a might play an important role in PCa development and progression.

In our study, an inhibitory effect of miRNA-302a on cell proliferation was observed in PCa PC-3 and DU145 cells, through hindering the G1 to S phase transition, which is considered as a key event during cell proliferation. In agreement with previous studies [8,10,14], we also found notable downregulation of *cyclin D1* in miRNA-302a overexpressing PCa cells. Interestingly, miRNA-302 is predicted to target many cell cycle regulators. For instance, Lin et al. demonstrated that miRNA-302 simultaneously suppressed both *cyclin E-CDK2* and *cyclin D-CDK4/6* signaling pathways [13], and this target blocking was regulated by several transcriptional factors, such as *Oct4* and *Sox2* [8]. However, Card et al. reported that miRNA-302a promoted an increase in S phase and a decrease in G1 phase in hESCs, although *cyclin D1* was also repressed [8]. Clearly, further research elucidating the exact mechanisms of miRNA-302 function is needed.

Our observations that overexpression of miRNA-302a in PCa cells induced cell growth inhibition in vitro and in vivo suggest that miRNA-302a might post-transcriptionally regulate a pivotal gene that is involved in cell proliferation. As an important oncogene, *AKT* influences a wide range of physiological functions, including metabolism, proliferation, survival, angiogenesis, migration, and invasion [16]. Likewise, *AKT* was proven to drive PCa formation in vivo [17]. Our results indicate that miRNA-302a suppressed the proliferation and tumorigenicity of PCa cells through the *AKT-GSK3β-cyclin D1* and the *AKT-p27* ^*Kip1*^ pathway, and by directly binding the 3΄UTR of *AKT*. Furthermore, the expression of *PI3K*, which is principal upstream effector of *AKT* and has also proven important in PCa development, were not affected by miRNA-302. The regulatory role of miRNA-302 in *AKT* was also demonstrated by Cai et al: after miRNA-302s transfection into cervical cancer cells, they observed elevated expression of cyclin-dependent kinase inhibitors *p27* ^*Kip1*^ and *p21* ^*Cip1*^, along with downregulated *AKT* levels. Furthermore, they also demonstrated that *cyclin D1* is another target gene of miRNA-302s, which accounted for our observation that cyclin D1 expression was not rescued after AKT restoration [10]. Hence, as a target of miRNA-302, *AKT* was notably suppressed and evoked alterations in many downstream signaling pathways.

Our findings also give certain clues with respect to miRNAs-targeted cancer treatment. Previous studies revealed that some specific miRNAs were often overexpressed in tumors, while most miRNAs were downregulated [18,19]. Global miRNA suppression was found to boost carcinogenesis in both in vitro and in vivo models [20], highlighting the protumorigenic effects following miRNA loss-of-function. Liang et al. observed a sensitizing role of miRNA-302 replacement therapy in breast cancer cells to ionizing radiation [21]. Another recent study reported that viral delivery of let-7 miRNA could inhibit tumor growth in a mouse lung adenocarcinoma model [22]. Likewise, our results validated the inhibitory effect of miRNA-302a replacement in PCa cells. Taken together, these studies suggest that overexpression of even a single miRNA in cancer cells might confer substantial therapeutic benefit.

In summary, our study demonstrates that miRNA-302a is pivotal for PCa cell growth by regulating G1-S phase transition. MiRNA-302a expression is suppressed in PCa tissues, and is even lower in PCa tissues with higher GS. Additionally, through direct binding to its 3΄UTR, miRNA-302a inhibits *AKT* expression, resulting in subsequent alterations in *AKT-GSK3β-cyclin D1* and *AKT-p27* ^*Kip1*^ pathways. Although it is clear that miRNA-302a participates in PCa, further studies are required to explain the precise mechanisms underlying its role in PCa progression, and thus determine its potential value as a biomarker and/or target of therapeutic intervention.

## Supporting Information

S1 Table Demographic and clinicopathological characteristics of 44 patients with prostate cancer (PCa). (DOCX)
