# Correction: MicroRNA-302a Suppresses Tumor Cell Proliferation by Inhibiting *AKT* in Prostate Cancer

**DOI:** 10.1371/journal.pone.0241462

**Published:** 2020-10-22

**Authors:** The PLOS ONE Editors

After this article [1] was published, concerns were raised about overlap in content between this article and another article that was published in *PLOS ONE* by different authors and retracted in 2015 [2, 3]. There is substantial overlap in text, and multiple figures appear similar or identical across the two articles although labels and legends indicate that the data were obtained from different cell and tissue types (colon cancer cell lines and tissues in the retracted article [2] versus prostate cancer cell lines and tissues in [1]).

The *PLOS ONE* Editors conducted follow up enquiries in response to the related article concerns.

The authors of [1] provided *PLOS ONE* with raw image files and underlying data for this study, as well as approval documents issued by the Animal Studies Ethics Committee of Fudan University Medical School and the Ethics Committee of Fudan University Shanghai Cancer Center. In addition, the head of the Division of Scientific Research, Shanghai Cancer Center, Fudan University, confirmed the research and submission history reported by the authors for [1] based on the outcome of a prior investigation by Fudan University.

Upon following up with the authors of the other article [2], the *PLOS ONE* Editors were unable to verify the integrity of that work, and as a result that article was retracted [3].

In light of the above information and documentation received from the authors of [1], the *PLOS ONE* Editors consider the related manuscript issue as resolved.

The ethics approval documents provided in post-publication discussions indicated that the names of approving committees were incorrectly listed in the published Ethics Statement for this article [1]. The Ethics Statement is updated to:

The use of patient samples was approved by the Ethics Committee of Fudan University Shanghai Cancer Center and signed informed consent was obtained from all study participants. The animal experiments were approved by the Animal Studies Ethics Committee of Fudan University Medical School.

In addition, the published Data Availability Statement for this article is incorrect. The statement is updated to:

All original underlying data for the study are available and will be provided upon request.
