# Supplementary material for: MicroRNA-302a Suppresses Tumor Cell Proliferation by Inhibiting AKT in Prostate Cancer
Source: PLoS One. 2015 Apr 29;10(4):e0124410. doi: 10.1371/journal.pone.0124410 (PMC4414271; doi:10.1371/journal.pone.0124410)
Supplement: S1 Table — (DOCX) [file pone.0124410.s001.docx]

**Table S1 Demographic and clinicopathological characteristics of 44 patients with prostate cancer (PCa)**

| clinicopathological parameters | n | miRNA-302a expression | P value |
| --- | --- | --- | --- |
|  |  | in PCa tissues |  |
| Age (y) |  |  |  |
| ＜65 | 16 | 0.0009±0.002 | 0.196 |
| ≥65 | 28 | 0.0038±0.008 |  |
| PSA (ng/ml) |  |  |  |
| ＜10 | 9 | 0.0023±0.004 | 0.347 |
| 10-20 | 8 | 0.006±0.011 |  |
| ＞20 | 27 | 0.0019±0.006 |  |
| T stage |  |  |  |
| T1 | 2 | 0.0004±0.0003 | 0.747 |
| T2 | 34 | 0.0025±0.006 |  |
| T3 | 8 | 0.0043±0.011 |  |
| N stage |  |  |  |
| N0 | 35 | 0.0034±0.0079 | 0.231 |
| N1 | 9 | 0.0002±0.0002 |  |
| M stage |  |  |  |
| M0 | 38 | 0.0032±0.0076 | 0.338 |
| M1 | 6 | 0.0001±0.00009 |  |
| Gleason score |  |  | **0.031** |
| =7 | 23 | 0.00507±0.009 |  |
| ＞7 | 21 | 0.00054±0.0018 |  |
